# Real‐World Response of Cemiplimab Immunotherapy for Treating Locally Advanced or Metastatic Cutaneous Squamous Cell Carcinoma in a Tertiary UK Oncology Centre

**DOI:** 10.1155/jskc/8993856

**Published:** 2026-08-10

**Authors:** Sumbal Umar, Zarmeen Shahid, Micheal O’Cathail, M. Ananth Sivanandan

**Affiliations:** ^1^ Department of Oncology and Radiotherapy, Nottingham City Hospital, Nottingham University Hospitals NHS Trust, Nottingham, UK, nhs.uk; ^2^ School of Medicine, University of Nottingham, Nottingham, UK, nottingham.ac.uk

**Keywords:** cemiplimab, cutaneous squamous cell carcinoma, immunotherapy, non-melanoma skin cancer

## Abstract

**Introduction:**

Cemiplimab has been licensed for use in the United Kingdom as the sole immunotherapy agent for first‐line use in advanced cutaneous squamous cell carcinoma (a‐cSCC). Given that previous clinical trials leading to the approval of cemiplimab had relatively small patient cohorts with relatively short follow‐up periods, with a large proportion of patients who had received prior systemic treatment, this retrospective real‐world study sought to add further insight into survival and toxicity in patients who exclusively had received first‐line use of cemiplimab, as well as comparing response rates (RRs) based on primary site location.

**Methods:**

This retrospective study identified patients diagnosed with a‐cSCC between 01/08/2019 and 31/03/2024 who received cemiplimab as first‐line treatment at our institution. Data to enable response assessment and toxicity were collected and analyzed.

**Results:**

50 patients were identified with a mean age of 75 years (range: 44–94). 78% (*n* = 39) of the patients’ primary lesion originated from the head and neck (H&N) region, whilst the remaining 22% (*n* = 11) had a primary lesion from other areas. No patients had received prior systemic treatment for a‐cSCC. Overall, the disease control rate (DCR) was 74%, and the RR was 55.8% with complete response in 35% (*n* = 15), partial response in 21% (*n* = 9), and stable disease in 19% (*n* = 8). In patients with H&N primary site of disease, RR was 58.9% versus 27.2% in patients with non‐H&N primary site of disease, with a *p* value of 0.027. The overall mean PFS for the study population was 27.3 months (95% CI: 21.2–33.5 months), with the median PFS not reached for the study duration. The median PFS for patients with H&N primary site of disease was not reached as compared to the median PFS of 4 months (95% CI: 2.2–5.7 months) for patients with non‐H&N primary site of disease, with a *p* value of 0.049. The median OS for the study population was 34 months (95% CI: 15.4–52.5 months). Grade 3 toxicities were reported in 22% (*n* = 11) with no Grade 4 toxicites.

**Discussion:**

The use of cemiplimab as first‐line treatment in a‐cSCC demonstrates high efficacy and low rates of serious toxicity, which is especially reassuring in a patient cohort comprising of more elderly patients. The high complete RR seen in our patient cohort may reflect the exclusive first‐line use of cemiplimab in our study, as per now established real‐world practice in a‐cSCC. Higher RRs and PFS are seen in patients with head/neck primary site of disease vs. non head/neck primary site, which should be evaluated further in future clinical trials.

## 1. Background

Cutaneous squamous cell carcinoma (cSCC) is the second most common type of skin cancer, with an estimated 310% increase in incidence in almost the last 30 years [1]. The most important risk factors for the development of cSCC include exposure to ultraviolet (UV) radiation, immunosuppression, and increasing age [2]; the risk increases significantly after the age of 75 [2]. Immunosuppression, whether acquired, such as from organ transplantation and human immunodeficiency virus (HIV) infection, or inherited, is associated with a 65‐250‐fold increased risk of development of cSCC [2]. Exposure to UV radiation, particularly UVB, is the most significant environmental risk factor for the development of cSCC, with UVB radiation causing direct DNA damage by formation of dipyrimidine dimers leading to mutations and carcinogenesis [3]. UVA radiation, on the other hand, causes formation of free radicals leading to indirect DNA damage [3].

Localized cSCC can be treated readily with surgery and/or radiotherapy, with a resulting good prognosis. Systemic therapy is currently reserved for locally advanced and metastatic cancers not amenable to radical surgery or radical radiotherapy. Prior to the use of immunotherapy, cytotoxic chemotherapy was the only drug option available in the United Kingdom for advanced cSCC (a‐cSCC) (defined as locally advanced or metastatic) and was associated with low efficacy and significant toxicities. Consequently, in many a‐cSCC patients who are often older with comorbidities, chemotherapy use was often limited [4, 5]. Historically, prognosis for patients with a‐cSCC has been poor, with an estimated median survival of 8–15 months [6]. With the advent of immune checkpoint inhibitors, such as cemiplimab, improved therapeutic outcomes have been witnessed in a‐cSCC management [7]. The improved outcomes seen with immunotherapy in a‐cSCC could be explained by the high tumor mutational burden seen in cSCC secondary to UV‐related mutagenesis [8]; the resulting high expression of neo‐antigens is hypothesized to increase chances for T‐cell recognition and clinically correlates with better immunotherapy outcomes [9].

Cemiplimab is a monoclonal antibody that targets the Programmed cell death 1 (PD‐1) receptor, thereby increasing the activity of the immune system against cancer cells. The initial trials evaluating cemiplimab in a‐cSCC patients, in which a large proportion had received prior systemic treatment, showed a response rate (RR) of approximately 47% and a Grade 3 adverse event rate of 42% [7]. NICE approved the use of cemiplimab in the United Kingdom via the Cancer Drugs Fund as a first‐line treatment for a‐cSCC in 2019 [10].

Approval of cemiplimab was based on the results of clinical trials with relatively small patient cohorts and relatively short follow‐up periods [11]. Therefore, studies of real‐world data are particularly pertinent in this setting to demonstrate further insights to assist clinical practice and decision‐making; for instance, data on RRs in a‐cSCC patients who have received no prior systemic treatment and RRs based on primary site would be of assistance. This retrospective monoinstitutional study conducted at a tertiary UK oncology center aimed to assess the real‐world effectiveness and safety of cemiplimab in the first‐line setting for a‐cSCC through evaluation of disease control rate (DCR), RR, survival outcomes including median overall survival (mOS) and median progression‐free survival (mPFS), and incidence of Grade 3 adverse events.

## 2. Methods

This study identified patients diagnosed with a‐cSCC between 01/08/2019 and 31/03/2024 who received cemiplimab as first‐line treatment at Nottingham University Hospitals NHS Trust, UK. This included patients with locally advanced and cSCC not amenable to radical surgery or radical radiotherapy.

This study was deemed to not present a risk to patient safety or patient data protection by the Nottingham University Hospital audit and governance department. As this study formed part of a local service audit, no further formal ethics review was deemed necessary in keeping with appropriate published guidelines [12]. As this was a retrospective observational study, individual informed consent was not required in accordance with institutional policy and applicable ethical guidelines.

All patients had been discussed prior to starting cemiplimab at the Nottingham specialist skin cancer multidisciplinary team (SSMDT) meeting, made up of specialists from dermatology, plastic surgery, clinical oncology, medical oncology, histopathology, and radiology, to confirm eligibility for cemiplimab and to decide that patients were not appropriate for radical surgery or radical radiotherapy. All patients were deemed eligible for cemiplimab treatment following discussion in this SSMDT, and Cancer Drugs Funding criteria were followed as per national specifications.

Inclusion criteria for this study were that patients were started on cemiplimab in the first‐line setting, had a histologically or cytologically confirmed diagnosis of cSCC, and must have received at least one dose of cemiplimab. Under these criteria, cemiplimab was continued until loss of clinical benefit, unacceptable toxicity, withdrawal of patient consent, or for a maximum treatment duration of 2 years (35 three‐weekly cycles), whichever occurred first. Treatment breaks of up to 12 weeks beyond the scheduled cycle length were permitted only to allow immune‐related toxicities to resolve. Elective discontinuation following complete response was not routinely undertaken because patients who discontinued treatment electively were not eligible for NHS‐funded rechallenge with cemiplimab in the event of subsequent disease recurrence.

Patients were excluded if they received cemiplimab beyond the first‐line setting, lacked histological or cytological confirmation of cSCC (a‐cSCC), had a non‐cSCC (e.g., vulval SCC), or did not receive at least one dose of cemiplimab.

Data were collected on variables including age, gender, performance status (PS), primary site of cSCC, whether they had locally advanced or metastatic disease, previous treatment history, and number of cycles of cemiplimab received. Response was determined using clinical assessment with examination for clinically assessable lesions and radiologically by cross‐sectional imaging where appropriate, based on Response Evaluation Criteria in Solid Tumors (RECIST), Version 1.1 [13].

The safety profile of cemiplimab was assessed retrospectively using Common Terminology Criteria for Adverse Events (CTCAE) V5 with respect to toxicities of Grade 3 or higher.

Patients were followed from the initiation of first‐line cemiplimab until death or the study data cutoff date (31 March 2024), whichever occurred first. Patients who remained alive at the data cutoff were censored on 31 March 2024.

For statistical analysis, DCR was calculated as the number of patients with stable disease, partial response, or complete response, based on either clinical or radiological criteria, divided by the total number of the studied population. RR was calculated as the number of patients with either partial response or complete response, based on either clinical or radiological criteria, divided by the total number of the studied population. Chi‐square analysis was used to calculate *p* value for comparison of RR and DCR between head and neck (H&N) primary site of disease versus non‐H&N primary site of disease. Survival outcomes including mPFS and mOS were calculated using Kaplan–Meier survival curves. The log‐rank test was used to calculate confidence intervals for mPFS and mOS and the associated *p* values. Cox regression analysis was used to determine hazard ratios and associated *p* values. Grade 3 adverse events leading to either temporary or permanent discontinuation of cemiplimab were recorded.

## 3. Results

### 3.1. Study Population Characteristics

Characteristics of the study population are summarized in Table 1. This study consisted of 50 patients with a median age of 75 years (range: 44–94 years). The majority of the study population (82%) were aged 65 years or older at the time of diagnosis of a‐cSCC. Males comprised 82% (*n* = 41) of the study population, whilst females were 18% (*n* = 9). PS was 1 in 70% (*n* = 35), 2 in 12% (*n* = 6), and 0 in 18% (*n* = 9) of the study population. 58% (*n* = 29) of the study population with a‐cSCC had locally advanced disease, and 42% (*n* = 21) had metastatic disease. 78% (*n* = 39) of the patients had a primary lesion originating from the H&N region, whilst the remaining 22% (*n* = 11) had a primary lesion from other areas. None of the study patients had received prior systemic treatment for a‐cSCC. The minimum number of cemiplimab cycles received was 1, whereas the maximum number of cycles received was 35. The median follow‐up from commencement of cemiplimab was 15.9 months (range: 0.9–54.8 months).

**TABLE 1:** Characteristics of the study population.

Variable		Frequency (%)
Mean age		75 Range: 44–94

Age ≥ 65		35 (82%)

Gender	Male	41 (82%)
	Female	9 (18%)

Performance status	0	9 (18%)
	1	35 (70%)
	2	6 (12%)

H&N primary site of disease		39 (78%)

Non‐H&N primary site of disease	Trunk	5 (10%)
	Hand–upper limb	3 (6%)
	Foot–lower limb	1 (2%)
	Unknown primary	2 (4%)

Prior systemic therapy		0

Minimum number of cemiplimab cycles received		1

Maximum number of cemiplimab cycles received		35

Median follow‐up	15.9 months	Range: 0.9–54.8 months

### 3.2. Treatment Efficacy

**FIGURE 1:**
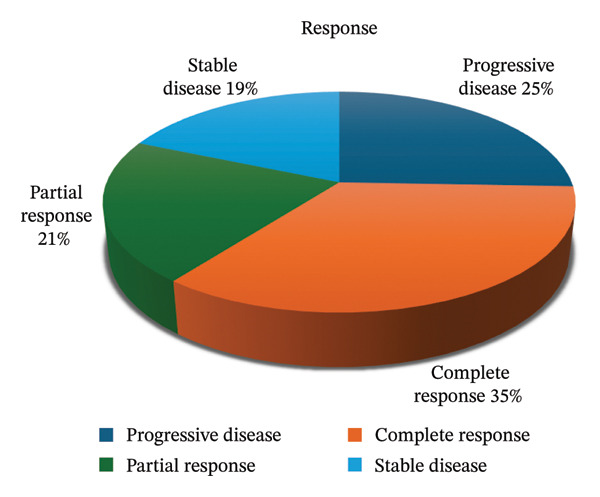
Overall response to cemiplimab in advanced cutaneous squamous cell carcinoma.

Overall, the DCR was 74%, and the overall RR (ORR) was 55.8%, with complete response in 35% (*n* = 15), partial response in 21% (*n* = 9), and stable disease in 19% (*n* = 8) over the study period (Figure 1).

**FIGURE 2:**
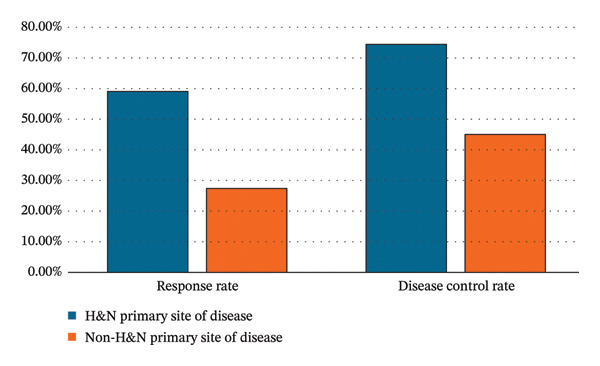
Comparison of DCR and ORR to cemiplimab in H&N vs. non‐H&N primary site of disease in advanced cutaneous squamous cell carcinoma.

In patients with H&N primary site of disease, ORR was 58.9% versus 27.2% in patients with non‐H&N primary site of disease, with a *p* value of 0.027 (Figure 2; Table 2). DCR in patients with H&N primary site of disease was 74.3% vs. 45.4% in patients with non‐H&N primary site of disease with *p* value of 0.234 (Figure 2; Table 2).

**TABLE 2:** Analysis of a‐cSCC with H&N primary site of disease vs. non‐H&N primary site of disease.

	Advanced cSCC with H&N primary (*n* = 39) (%)	*p* value	Advanced cSCC with non‐H&N primary (*n* = 11) (%)
Response rate	**58.9**	0.027	**27.2**
Disease control rate	**74.3**	0.234	**45.4**

*Note:* The bold values (58.9, 74.3, 27.2, and 45.4) represent percentages (%) for response rate and disease control rate in the respective patient groups.

**FIGURE 3:**
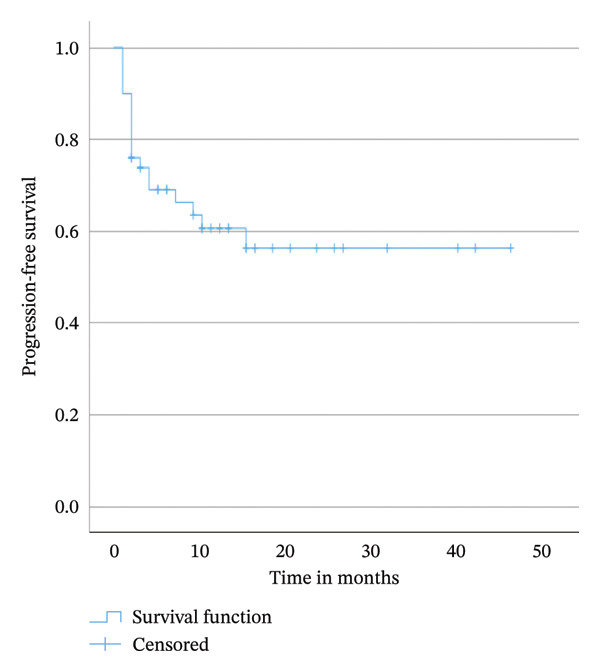
Kaplan–Meier graph of overall progression‐free survival (PFS) in advanced cutaneous squamous cell carcinoma.

The overall mean PFS for the study population was 27.3 months (95% CI: 21.2–33.5 months), with the median PFS not reached for the study duration (Figure 3).

**FIGURE 4:**
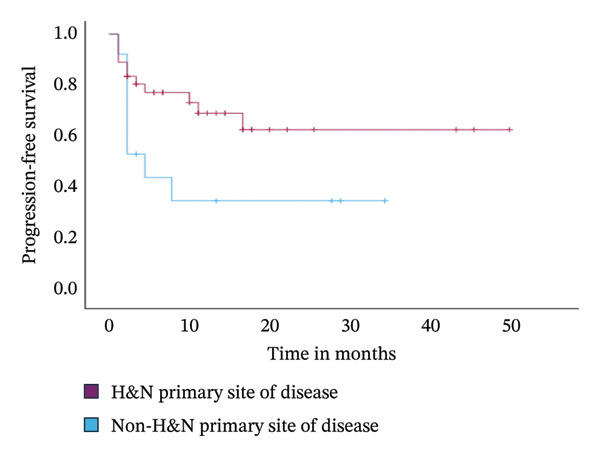
Median progression‐free survival H&N primary site of disease vs. non‐H&N primary site of disease in advanced cutaneous squamous cell carcinoma.

The median PFS for patients with H&N primary site of disease was not reached as compared to the median PFS of 4 months (95% CI: 2.2–5.7 months) for patients with non‐H&N primary site of disease, with a *p* value of 0.049 (Figure 4).

Regarding PFS between the two groups, the hazard ratio was 0.426 for patients with H&N primary site of disease (95% CI: 0.170–1.063) as compared to patients with non‐H&N primary site of disease, with a *p* value of 0.067 (Table 3).

**TABLE 3:** Cox regression analysis of progression‐free survival H&N primary site of disease vs. non‐H&N primary site of disease in advanced cutaneous squamous cell carcinoma.

Hazard ratio	0.426
Confidence interval	0.170–1.063
*p* value	0.067

**FIGURE 5:**
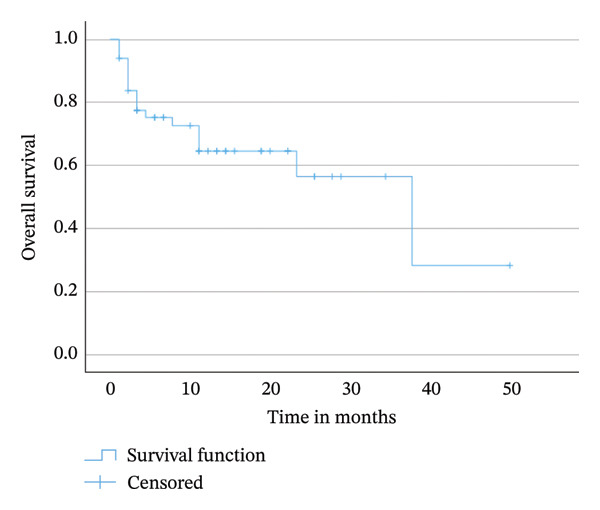
Kaplan–Meier graph of overall survival (OS) in advanced cutaneous squamous cell carcinoma.

The median OS for the study population was 34 months (95% CI: 15.4–52.5 months) (Figure 5).

### 3.3. Treatment‐Related Toxicity

Grade 3 toxicities were reported in 22% (*n* = 11), including autoimmune colitis 6% (*n* = 3), widespread maculopapular rash 4% (*n* = 2) autoimmune nephritis 2% (*n* = 1), autoimmune hepatitis 2% (*n* = 1), reactive arthritis 2% (*n* = 1), autoimmune endocrinopathy 2% (*n* = 1), and autoimmune hematological toxicity 4% (*n* = 2) (Table 4). Cemiplimab treatment was permanently discontinued in all these patients who developed Grade 3 toxicities. Grade 4 toxicities were not reported in the study population.

**TABLE 4:** Grade 3 adverse events associated with cemiplimab.

Grade 3 adverse events	*N* = 11 (22%)
Autoimmune colitis	*N* = 3 (6%)
Widespread maculopapular rash	*N* = 2 (4%)
Autoimmune hematological toxicity	*N* = 2 (4%)
Autoimmune nephritis	*N* = 1 (2%)
Autoimmune hepatitis	*N* = 1 (2%)
Reactive arthritis	*N* = 1 (2%)
Autoimmune endocrinopathy	*N* = 1 (2%)

## 4. Discussion

This retrospective monoinstitutional study confirms the real‐world safety and effectiveness of cemiplimab in a‐cSCC in the first‐line setting over a prolonged follow‐up period.

The RRs and serious (Grade 3) toxicity rates in our patient cohort are comparable to the initial clinical trials and recently published real‐world data [14]. Interestingly, the complete RR in our patient cohort was high at 35%; this may be associated with the exclusive use of cemiplimab in the first‐line setting in our study, whereas a significant proportion of patients in the initial trials had received prior systemic treatment.

We found that those patients with H&N primary site of the disease had a significantly higher mPFS and RR than those patients with non‐H&N disease. This is the first study that we are aware of that demonstrates that, for patients in which cemiplimab has been used in the noncurative setting, a‐cSCC from a H&N primary site of the disease has a statistically significant higher RR to immunotherapy vs. non‐H&N primary site of disease. Therefore, this is indicative that cemiplimab may have higher therapeutic efficacy in patients whose a‐cSCC is from a H&N primary site. The H&N region is the most UV‐exposed region of the body, which would lead to tumors in this region with higher mutational burden [8, 9]. This could explain the higher efficacy of immunotherapy in the H&N primary site of a‐cSCC, as studies have shown improved RRs to immunotherapy in tumors with high mutational burden [8]. This study does not have data on tumor mutational burden or PDL1 expression, as it is not a standard NHS practice for a‐cSCC.

Furthermore, a numerical difference in DCR was also observed between the subgroups; however, this did not achieve statistical significance. Consequently, no definitive conclusions can be drawn regarding differences in DCR based on these findings. The observed trend should be interpreted cautiously and may serve to inform future studies with larger sample sizes.

It is reassuring that, given the majority of patients in our patient cohort are over 65, no Grade 4 or 5 toxicity was observed. Grade 3 toxicity rates in our patient cohort were similar to initial studies [11], with treatment discontinuation rates from toxicity being comparable to other real‐world published series [14]. This confirms that, for the majority of patients with a‐cSCC, immunotherapy represents a tolerable treatment. We recognize discontinuation rates appear higher in real‐world populations, as compared to trial populations, likely due to differences in patient characteristics between trial and real‐world populations, including preexisting comorbidities. Higher rates of treatment discontinuation have been found in real‐world patients as compared to trial patients in other oncology studies [15, 16].

The prolonged follow‐up period of our study provides additional reassurance that no new safety signals were seen with immunotherapy in a‐cSCC patients. Considering the long follow‐up period, our study demonstrates that in those patients with H&N primary, median PFS was not reached. This suggests that patients with H&N primary are more likely to achieve durable responses associated with more prolonged survival. Patients with H&N primary represented the majority of our patient cohort, as per other studies [11, 14]; therefore, these survival data are of pertinence. Longer‐term survival, and even potential cure, is seen in other tumor types with advanced disease with immunotherapy, including melanoma [17] and non‐small cell lung cancer [18]. Our data suggest this may be applicable to patients with a‐cSCC, particularly those with a H&N primary.

### 4.1. Limitations of Study

There are some potential limitations of the study which can impact the reliability and generalizability of the findings. This includes the retrospective nature of the study, so data were not standardized as would be seen in a prospective trial. Clinical examination response, radiological response, and toxicity data were obtained from real‐world patient records, so results were dependent on completeness and accuracy ascertained from these records. Confounding factors could not be accounted for in this study. Other limitations of the study could be variability in timing of scans for response assessment, as well as a small patient population, which makes it difficult to make definite conclusions from the study. Pathological confirmation of complete response with repeat biopsy is not a standard clinical practice in the United Kingdom for patients receiving palliative treatment. This represents a key limitation in interpreting response outcomes, as incongruities can exist between complete response and pathological response; for instance, a complete clinical response could be seen despite microscopic disease still remaining. In addition, only grade ≥ 3 treatment‐related adverse events were systematically collected. As Grades 1‐2 toxicities were not captured, the overall tolerability profile of cemiplimab may be incompletely characterized in this study.

## 5. Conclusion

Our monoinstitutional retrospective study adds to the current growing evidence supporting the use of cemiplimab as first‐line treatment in a‐cSCC with high efficacy and low rates of serious toxicity [19, 20], which is especially reassuring in a patient cohort comprising of more elderly patients. We have highlighted the importance of tumor location on RR and survival outcomes as evidenced by improved outcomes in patients with head/neck primary site of disease vs. non head/neck primary site; this warrants evaluation in future trials. We eagerly await publication of long‐term survival data in patients with a‐cSCC treated with immunotherapy.

## Funding

No funding was received for this study.

## Conflicts of Interest

The authors declare no conflicts of interest.

## Data Availability

Research data are not shared.
